# Impact of planting density and soaking seeds in melatonin solution on yield, secondary products content and antimicrobial activity of lovage plant

**DOI:** 10.1016/j.sjbs.2021.12.048

**Published:** 2021-12-23

**Authors:** Amira K.G. Atteya, Aishah N. Albalawi, Hala M. Bayomy, El Moataz Bellah A. El-Naggar, Mahmoud H. Ghozlan, Esmail A.E. Genaidy

**Affiliations:** a Horticulture Department, Faculty of Agriculture, Damanhour University, Damanhour 22516, Egypt; b Department of Analytical Chemistry, Tabuk University, University College of Haql, Tabuk 71491, Saudi Arabia; c Department of Nutrition and Food Science, Tabuk University, Tabuk 71491, Saudi Arabia; d Department of Food Science and Technology, Damanhour University, Damanhour 22516, Egypt; e Pharmacognosy Department, Faculty of Pharmacy, Damanhour University, Damanhour 22516, Egypt; f Plant Pathology Department, Faculty of Agriculture, Damanhour University, Damanhour 22516, Egypt; g Pomology Department, National Research Centre, Giza 12622, Egypt

**Keywords:** MBC, minimal bactericidal concentration, MFC, minimal fungicidal concentration, Lovage, Planting density, Melatonin, Essential oil analysis, Total phenolic and antioxidant content, Antimicrobial activity

## Abstract

Many studies worldwide have been done on the effect of medicinal uses of lovage plant but, very little works have been done on its production. In this study the effect of different planting density and soaking seeds in different concentration of melatonin solution as well as their combination treatments on yield, secondary products content and antimicrobial activity of lovage plant were studied. It was observed that using planting space of 15 cm gave the maximum mean values of total phenolic and antioxidant content and essential oil percentage. Using 30 cm planting space gave the maximum mean values of plant height, yield of herb fresh and dry weight per hectare, yield of roots dry weight and essential oil per hectare. While the plant space of 45 cm recorded the maximum mean values of fresh and dry weight of herb and roots fresh weight per plant and chlorophyll content. For melatonin levels, using 100 µM melatonin solution had the minimum mean values of number of days to emergence. While, soaking seeds in 75 µM melatonin solution recorded the best results of all studied parameters. Regarding the combination treatments, measurements comprising of herb fresh and dry weight as well as essential oil yield per hectare showed that the combination treatment of 30 cm between plants in row plus soaking seeds in 75 µM melatonin solution was able to achieve the maximum values of these parameters. While the combination treatment of 15 cm between plants in row plus soaking solution of 75 µM melatonin is recommended for getting the maximum yield of root fresh and dry weight per hectare and the maximum total phenolic and antioxidant contents per herb in both cuts of both studied season. The first major compound of lovage essential oil of herb is α-terpinyle acetate followed by β-Phellandrene. The percentages of these compounds were affected by the applied treatments. The volatile oil of lovage plant exhibits high antibacterial and antifungal properties in the concentrations range of 75–100 µg mL^−1^.

## 1 Introduction

*Levisticum officinale* Koch (lovage) is a perennial plant belongs to family apiaceae. It is a robust, glabrous, perennial herbaceous plant which grows up to 2–2.5 m. It is native to Southwest Asia and southern Europe (Tutin et al., 1968). The leaves are 2–3 pinnate, large and bright green. The flowers are yellowish, the fruits are 5–7 mm long and broad elliptic and the 1000 seed weight is 3.64 g (Heeger, 1956). The main producers are Germany, Hungary, the Netherlands, Poland and Belgium. The traditional usage of lovage in several diseases has been documented in previous resources and in folk tradition. Lovage exhibits diverse pharmacological activities, containing estrogenic, apoptotic and antimycobacterial activities (Bogucka-Kocka et al., 2008, Schinkovitz et al., 2008). It's used to treat kidney stones, jaundice, irritable bowel syndrome, malaria, sore throat, tonsillitis, and cystitis, as well as rheumatism, gout, boils, and eye inflammation. The bioactivity of lovage oil, and dosages of 40 ppm were found to be effective in anticancer studies. It is not advised for pregnant women because it has been linked to the onset of menstruation. People with kidney illness should avoid using this plant because of its irritating impact, which can cause kidney damage in high dosages . Lovage plants contain a volatile oil, angelic acid, a bitter extractive, resins, etc. (Simon et al., 1984, Karnick, 1994). A lot of phytochemical studies have been done to determine the chemical composition of the essential oil of lovage. The chemical composition of different extracts of lovage revealed more than 190 volatile compounds, frequently monoterpenes and phtalide (Musitelli and Bossi, 2014). β-phellandrene, α-terpinyl acetate and Zligustilide are the main components of lovage oil that present in different value in the various plant organs. It seemed that, the content and composition of the essential oil in lovage plant is determined by many factors. According to Samiee et al. (2006) the main constituents in the oil of lovage, were α-terpinyl acetate (40.5%) and β-phellandrene (16.7%) were the major components. The main components of the oil in the other report were ß-phellandrene (42.5%), cc-terpineol (27.9%), cis-ocimene (7.5%) (Reza and Abbas, 2007). All these finding emphasis on one fact that growth of plants and their content of bioactive compounds depend on many factors, especially genetic, physiological and environmental factors. For example, the introduction of new crops to cultivate in new areas other than their habitat enrich the flora of these areas and give different chemical composition of these new imported plants under the new environmental conditions which will be suitable for local and international uses. Meanwhile, the first step for establishing cultivation of import plants such as lovage under the new environmental conditions is finding the best treatments of agricultural practices to maximizing their production under this new location.

Planting density has a great impact on the yield of plants. Following the optimum density, the highest productivity per unit of area will be achieved. Where the optimum density works to organize and facilitate other agricultural practices and regulate competition between plants over available natural resources such as solar radiation (Casper and Jackson, 1997, Sharratt and Mcwilliams, 2005, Xu et al., 2008), content of elements, moisture and oxygen in the soil, as well as the available soil area for growth of roots (Dong et al., 2010, Ren et al., 2017, Liu et al., 2019). Therefore, the efficiency of the photosynthesis process will be increased, which is reflected on the productivity of all different parts of the plant. This optimum density varies according to the type of plant and soil, the other agricultural practices as well as the purpose of production. Many researchers such as Xu et al. (2008) on maize, Rahman and Hossain (2011) on soybean, Ren et al., 2017, Liu et al., 2019, Cong et al., 2019, Tian et al., 2020 on oilseed rape and Jingang et al. (2019) on sunflower found that growth and yield of plants were improved with following the optimum planting density.

As a result of reducing the period between planting and emergence of seeds, the produced seedlings become more strong (Fenner and Thompson, 2005). As the long period of emergence expose the seeds to some environmental and microbial hazards of the soil, such as lack of sufficient moisture, light and oxygen, as well as attacking micro-organisms. Lovage seeds are characterized by a low emergence rate of about 24.67%, as well as the presence of a dormancy percentage with them (Khodashenas et al., 2015). This phenomenon of embryo that is not fully developed in most Apiaceae family (Martin, 1946) has been studied in *Apium graveolens* (Pressman et al., 1977) and *Conium maculatum* (Baskin and Baskin, 1990). Where the embryo needs appropriate conditions of temperature, humidity, hormones and light to begin in growth and emergence. Therefore, the soaking of seeds before planting in melatonin is appropriate treatment for accelerate the growth of the embryo, which will be reflected on the productivity of the cultivated plants. Melatonin is a small molecule hormone found in plants and animals. It is a broad-spectrum growth regulator and antioxidant (Haoshuang et al., 2019). It enhances plant growth and development especially in abiotic stresses and acts as an osmotic regulatory substance in plants, enabling the maintenance of ion homeostasis and growth regulation (Timothy and Colmer, 2015). Previous studies have shown that exogenous melatonin treatments of spraying tea plants with 100 µM, soaking cucumber seeds in 0.1–500 µM, soaking tomato seedling roots in 1 μmol L^−1^, priming cotton seeds in 10, 20, 50 and 100 μM melatonin solution (Jiahao et al., 2019, Zhang et al., 2014, Li et al., 2016a, Li et al., 2016b, Chen et al., 2020) respectively improved growth under the condition of normal and abiotic stresses.

Due to the shortage of information about the response of lovage plant to agriculture practices in general and as a now import plant under the Egyptian environmental conditions particular, this study was done to reach the best practices including plant spacing and melatonin concentration as a soaking treatments of seeds to maximize productivity, essential oil, polyphenolics, antioxidant content, yield and antimicrobial activity of lovage Koch. plant worldwide and establishment its cultivation under the Egyptian environmental conditions as a new imported medicinal and aromatic plant to try to insert it into Egyptian flora for domestic use and export.

## 2 Materials and methods

The present investigation was carried out during two successive seasons of 2018 / 2019 and 2019 / 2020 in an open field of a private farm in Kom-Hamada, EL-Behira governorate, Egypt, to study the effect of plant spacing and soaking seeds in melatonin on growth as well as essential oil, polyphenolics, antioxidant content, yield and antimicrobial activity of lovage plants.

### 2.1 Plant material

Seeds of *Levisticum officinale* L. (lovage) plant were imported from Enza Zaden company in North Holland. They were cultivated on the October of 2018 and 2019. Pest control and other agricultural practices, such as irrigation, etc. were applied, wherever it was necessary and as commonly recommended. The environmental conditions of culture location according to Meteorological data from Central Lab. for Agricultural Climate, Agricultural Research Center, Ministry of Agriculture and Land Reclamation, Egypt are shown in the Table 1.

**Table 1:** The monthly mean values of the maximum, minimum air temperatures (C°), relative humidity (%), rain (mm/day) and day length (hour) in El-Behiera location during both seasons of the study.

Date	2018/2019					2019/2020				
	Temperature (°C)		Relative humidity %	Rain/(mm day)	Day Length (h)	Temperature (°C)		Relative humidity	Rain/(m day)	Day Length (h)
	Max	Min				Max	Min			
October	28.15	22.80	57.50	0.55	11:33	27.00	20.25	57.20	0.00	11:35
November	24.15	18.05	58.70	1.40	10:55	22.95	16.95	62.30	0.95	10:55
December	18.35	13.60	67.55	0.70	10:35	20.55	15.10	68.30	0.20	10:35
January	17.00	11.05	66.25	0.15	10:48	18.30	12.70	67.45	1.30	10:46
February	17.50	10.95	64.25	0.15	11:20	19.75	12.85	62.15	0.35	11:19
March	21.01	13.06	0.50	0.00	11:56	23.97	14.28	50.18	0.00	11:54

### 2.2 Treatment

The experiment laid out is a split plot arranged in Randomized Complete Block Design (RCBD) with three replications during October–March 2018/2019 and 2019/2020. The main plots assigned to plant spacing and the sub plots deviated to soaking seeds of lovage plants in melatonin. All possible combination of two studied factors were done (Table 2). It was taken two cuts of all treatments. The experiment included 12 treatments which were the combination between plant spacing (15, 30 and 45 cm) and soaking seeds before planting for 8 h in different concentration of melatonin (without soaking, 0, 75 and 100 µM). Melatonine (N-Acetyl-5-methoxytryptamine, C_13_H_16_N_2_O_2_) was brought from Sigma-Aldrich. The experimental unit area was 97.2 m^2^ with 13.5 m length and 7.2 m width and contained twelve rows. The distance between rows 60 cm apart and between plants in the same row were 15, 30 or 45 cm. Each main plot contained about 120, 60 or 40 plants under surface irrigation. Seeds were sown on 1st of October in the first and second seasons, respectively. Seeds (three seeds hill^−1^) were sown on one side of the row. After 26 days, the seedlings were thinned to one plant per hill. The plots were weeded every two weeks as it possible. The physical and chemical properties of the soil samples were determined according to Jackson, 1973, Cottenie et al., 1982 as shown in Table 3.

**Table 2:** All different used combinations treatments of plant spacing and melatonin concentrations with the following.

Treatments	
T1	15 cm between plants in row and without soaking (control).
T2	15 cm between plants in row and 0 µM melatonin soaking solution.
T3	15 cm between plants in row and 75 µM melatonin soaking solution.
T4	15 cm between plants in row and 100 µM melatonin soaking solution.
T5	30 cm between plants in row and without soaking.
T6	30 cm between plants in row and 0 µM melatonin soaking solution.
T7	30 cm between plants in row and 75 µM melatonin soaking solution.
T8	30 cm between plants in row and 100 µM melatonin soaking solution.
T9	45 cm between plants in row and without soaking.
T10	45 cm between plants in row and 0 µM melatonin soaking solution.
T11	45 cm between plants in row and 75 µM melatonin soaking solution.
T12	45 cm between plants in row and 100 µM melatonin soaking solution.

**Table 3:** The physical and chemical properties of the experimental soil.

Property	Unit	Value
Clay	%	34
Sand	%	27
Silt	%	39

Texture class		Clay loam
O.M.	%	1.4
pH	7.8	
ECe	ds m^−1^	2.3
CaCO^3^	%	1.8

*Soluble ions (meq L^−1^)*		
HCO^3–^	2.5	
Cl^-^	9.3	
SO^2-^	10.7	
Ca^2+^	10.9	
Mg ^2+^	6.02	
Na^+^	4.55	
K^+^	0.32	

### 2.3 The following data were recorded each season

#### 2.3.1 Emergence parameters

Three 30 cm diameter pots for every melatonin treatment were planted with soaking seeds (ten seeds per pot). Each pot content about 10 kg of field soil in an open field conditions. This separated stage take 20 day from planting until the end of it. As a completely randomized design (CRD) both of number of days until emergence (day) and emergence percent (%) were recorded.

#### 2.3.2 Growth and yield parameters

Plant height (cm) from the surface of the soil to the top of the plant, fresh and dry weight of herb per plant (g), yield of fresh weight of herb (ton ha^−1^) and dry weight of herb (kg ha^−1^) were measured at the end of every cut of every season. While the roots fresh and dry weight per plant (g) and per hectare (kg ha^−1^) were recorded at the end of every season. The drying of all plant parts was done by putting them in oven under 48 °C for three days until getting a constant weight.

#### 2.3.3 Total chlorophyll content (SPAD unit)

It was quantified using a SPAD-502 Chlorophyll Meter (Minolta Camera Co., Ramsey, NJ).

#### 2.3.4 Total phenolic (mg gallic1 g^−1^ herb)

It was determined according to the method of Singleton and Rossi (1965).

#### 2.3.5 Antioxidant content (mg TE 100 g^−1^ herb)

Free radical scavenging capacity of extracts was determined using the stable DPPH• according to Hwang and Do Thi (2014). The standard curve was prepared using Trolox. Results were expressed as mg Trolox equivalents (TE) 100 g^−1^ herb.

#### 2.3.6 Essential oil extraction

Essential oil of lovage were extracted from aerial parts of each treatment according to Guenther (1961) and as was mentioned in Atteya and El Gendy (2018) after the harvest in the end of every cut.

#### 2.3.7 GC–MS analysis

The essential oil of the selected treatments was carried out by GC–MS analysis as was mentioned in Atteya and El Gendy (2018).

#### 2.3.8 Simple correlation coefficients

Simple correlation coefficients among various studied characters of lovage in this study as affected with plant spacing and seeds soaking combination treatments in both seasons.

#### 2.3.9 Antimicrobial activity assays

For testing the anti-bacterial and anti-fungal effect of lovage Koch essential oil, the successive double dilution method was used. For this, the initial stage, 1 mL of peptone broth medium for tested bacteria was prepared and the following: phytopathogenic bacteria of *Ralstonia solanacearum*, *Pectobacterium Carotovorum* subsp atrosepticum and *Pectobacterium Carotovorum* subsp Carotovorum were examined. Potato dextrose broth was used as inoculation medium for the tested fungal strains i.e., *Fusarium roseum* Subsequently, for each bacterial and fungal isolates 1 mL of the obtained microbial suspension was dropped in a tube containing 9 mL of sterile distilled water. The content of the tube was mixed, after which 1 mL was transferred to the tube no. 2 of the 10-tube series containing 9 mL of sterile distilled water. The procedure was repeated until the tube no. 10 of the series. Thus, the concentration of the initial preparation decreased 2-fold in each subsequent tube. From the 5-th tube of the series were taken 0.1 mL of the microbial suspension, For the estimation of the minimal used concentration of essential oil produced from the treatment of 30 cm plant spacing and seeds soaking solution of 75 µM melatonin as bactericidal and fungicidal concentrations (MBC, MFC), the contents of the last test tubes were seeded on peptone Agar medium for determination of minimal bactericidal effect and Potato dextrose agar medium for minimal fungicidal effect. The seeded Petri dishes were kept in the incubator at 25 °C for 24–72 h. The concentration of the tested preparation that does not allow the growth of any colony of bacterial or fungal colony is considered to be the minimal bactericidal and fungicidal concentrations of the preparation. According to Wayne (1999).

### 2.4 Statistical analysis

The experiment laid out is a split plot arranged in Randomized Complete Block Design (RCBD) with three replications. Except for emergence test it was a completely randomized design (CRD). Analysis of variance with SAS software (SAS Institute Inc., 1988) was carried out on the all tested treatments data. Treatments’ means were compared using the LSD test at 5% level of probability. The experiment was repeated in the second year at the same site using the same steps and techniques of the first year to compare the results of the two cuts of the two successive seasons.

## 3 Results

### 3.1 Effect of melatonin on emergence parameters

Regarding mean values of soaking treatments, results in Table 4 indicate that the three tested treatments had significant effect on decreasing the number of days until emergence and increasing emergence percentage as compared with the control in both seasons. Moreover, the minimum number of days until emergence (5.13 and 6.67 day) was observed with the treatment of soaking seed in 100 µM melatonin and the maximum emergence percent (86.67 and 86.67 %) was found with 75 µM melatonin soaking solution before planting them in both seasons, respectively. From the other hand the maximum number of days to emergence (13.00 and 13.33 day) as well as the minimum emergence percent (56.67 and 53.33%) was noticed with control treatment (without soaking) in both seasons.

**Table 4:** The mean values of number of days to emergence (day) and emergence percent (%) of lovage seeds as affected with melatonin treatments in both seasons.

Concentrations (µM)	Number of days to emergence (day)		Emergence percent (%)	
	First season	Second season	First season	Second season
Without soaking	13.00 ± 1.00 a*	13.33 ± 0.58 a	56.67 ± 5.77 c	53.33 ± 11.55 c
0	8.93 ± 0.61b	8.33 ± 0.58 b	73.33 ± 5.42 b	73.33 ± 5.76 b
75	6.80 ± 0.80 c	7.33 ± 0.54 bc	86.67 ± 6.11 a	86.67 ± 5.81 a
100	5.13 ± 0.50 d	6.67 ± 0.57 c	76.67 ± 5.67 ab	80.00 ± 0.0 ab

* Means followed by the same letter are not significantly different by the protected LSD, P ≤ 0.05. Data are means value ± SE (n = 3).

### 3.2 Plant height

Experimental results in Table 5 show that planting density, melatonin concentration and the interaction of them were able to make significant differences in the height of lovage plants in the first and second seasons. Moreover, planting distance of 30 cm between plants in the same row gave the maximum plant height (36.5 and 28.9 cm) in the first season and (38.1 and 30.8 cm) in the second one for the both cuts, respectively compared with the other used planting spaces. In addition, soaking seeds in 75 µM melatonin had the highest lovage plants (35.9 and 35.8 cm in the first cut) and (27.7 and 29.1 cm in the second cut) in both seasons, respectively. As a result of that, the seventh combined treatment of planting space 30 cm plus 75 µM melatonin recorded the maximum plant height (38.2 and 30.1 cm in the first season and 39.4 and 32.6 cm in the second season) in the two cuts.

**Table 5:** The mean values of plant height (cm) of lovage plants as affected with plant spacing, melatonin concentrations and their combination treatments in both seasons.

Treatments	First season		Second season	
	1^st^ cut	2^nd^ cut	1^st^ cut	2^nd^ cut
*Planting space between plants in row (cm)*				
15	30.8 ± 3.0 c*	23.1 ± 1.8 b	30.3 ± 2.0 c	21.8 ± 2.4 c
30	36.5 ± 2.2 a	28.9 ± 1.7 a	38.1 ± 1.5 a	30.8 ± 2.6 a
45	34.0 ± 2.6 b	27.0 ± 1.8 a	35.0 ± 1.3 b	28.9 ± 1.9 b

*Melatonin concentrations (µM)*				
Without soaking	30.1 ± 3.0 c	24.6 ± 2.7 c	32.7 ± 4.2 c	24.7 ± 3.7 c
0	33.9 ± 3.0 b	26.3 ± 3.3 b	34.6 ± 2.7 b	27.0 ± 4.6 b
75	35.9 ± 2.1 a	27.7 ± 2.6 a	35.8 ± 3.4 a	29.1 ± 5.7 a
100	35.2 ± 2.9 ab	26.8 ± 2.8 ab	34.8 ± 3.9 b	27.8 ± 3.1 b

*Interaction*				
T1	26.7 ± 0.6 f	21.5 ± 1.8 h	27.7 ± 1.5 h	20.0 ± 1.0 e
T2	30.5 ± 2.2 e	22.7 ± 1.7gh	31.7 ± 0.3 fg	21.3 ± 1.0e
T3	33.8 ± 1.4 bcd	24.6 ± 1.1 efg	31.8 ± 0.8 fg	20.3 ± 0.3 e
T4	32.2 ± 1.0 de	23.8 ± 1.7 fgh	30.2 ± 1.4 g	25.6 ± 0.6 d
T5	33.3 ± 0.8 cd	26.3 ± 0.8 def	36.7 ± 1.8 bcd	27.0 ± 1.8 cd
T6	36.5 ± 1.2 ab	29.4 ± 0.8 abc	37.7 ± 1.3 abc	31.4 ± 0.9 ab
T7	38.2 ± 1.2 a	30.1 ± 1.0 a	39.4 ± 0.8 a	32.6 ± 0.8 a
T8	37.8 ± 1.5 a	29.8 ± 0.7 ab	38.8 ± 1.2 ab	32.1 ± 1.4 a
T9	30.3 ± 1.2 e	25.8 ± 1.9 def	33.9 ± 0.4 ef	27.0 ± 1.5 cd
T10	34.7 ± 1.2 bcd	26.7 ± 2.6 cdef	34.4 ± 1.3 de	28.2 ± 1.4 c
T11	35.7 ± 0.6 abc	28.5 ± 0.5 abcd	36.2 ± 1.1 cd	31.0 ± 1.0 ab
T12	35.5 ± 2.3 abc	27.0 ± 1.0 bcde	35.3 ± 1.0 de	29.3 ± 1.1 bc

* Means followed by the same letter are not significantly different by the protected LSD, P ≤ 0.05. Data are means value ± SE (*n* = 3). 1st cut and 2nd cut means the first and second cutts, respectively.

### 3.3 Herb fresh weight

Herb fresh weight per plant and per hectare of lovage varied according to planting space as well as soaking seed treatments and their interaction. Data in Table 6, Table 7 indicates that despite the planting at 45 cm distance between plants in the same row gave the heaviest herb fresh weight per plant (258 and 447 g plant^−1^ in the first season and 209 and 387 g plant^−1^ in the second season, respectively), the maximum yield per hectare (12.1 and 20.4 ton ha^−1^ in the first season and 10.0 and 18.3 ton ha^−1^ in the second season, respectively) was found with planting distance of 30 cm between plants in the same row compared with the other planting space treatments in the first and second cuts. Soaking solution of 75 µM melatonin gave the highest response to the herb fresh weight in the first season (199 and 353 g plant^−1^ and 11.3 and 20.0 ton ha^−1^) and during the second season (163 and 312 g plant^−1^ and 9.2 and 17.3 ton ha^−1^) in the first and second cuts, respectively compared with the other melatonin concentrations. Despite planting at distance 45 cm plus soaking seeds 75 µM melatonin solution recorded the maximum herb fresh weight per plant (276 and 474 g plant^−1^ in the first season and 221 and 434 g plant^−1^ in the second one) for the two cuts, respectively the maximum herb fresh weight per hectare (12.6 and 22.9 ton ha^−1^ in the first season and 10.4 and 19.8 ton ha^−1^ in the second season) for the both cuts, respectively was found with the treatment of 30 cm between plants in the same row plus 75 µM melatonin for soaking seeds compared with the other combinations treatments.

**Table 6:** The mean values of herb fresh weight (g plant^−1^) of lovage plants as affected with plant spacing, melatonin concentrations and their combination treatments in two cuts of both seasons.

Treatments	First season		Second season	
	1^st^ cut	2^nd^ cut	1^st^ cut	2^nd^ cut
*Planting space between plants in row (cm)*				
15	85 ± 8.1 c*	145 ± 23.9 c	73 ± 6.9 c	128 ± 13.7 c
30	218 ± 9.1 b	367 ± 38.0 b	180 ± 4.6 b	330 ± 21.8 b
45	258 ± 12.5 a	447 ± 18.8 a	209 ± 9.9 a	387 ± 30.4 a

*Melatonin concentrations (µM)*				
Without soaking	176 ± 76.5 d	288 ± 138.3 d	148 ± 62.8 c	256 ± 112.6 c
0	183 ± 78.6 c	305 ± 134.2 c	152 ± 61.5 cb	279 ± 114.7 b
75	199 ± 81.5 a	353 ± 136.5 a	163 ± 62.4 a	312 ± 129.6 a
100	190 ± 77.5 b	332 ± 135.2 b	155 ± 62.2 b	278 ± 115.0 b

*Interaction*				
T1	77 ± 7.7 i	114 ± 3.5 k	65 ± 3.9 f	110 ± 5.8 i
T2	81 ± 3.8 hi	135 ± 3.1 j	72 ± 1.7 f	128 ± 6.3 h
T3	94 ± 1.9 g	175 ± 0.8 h	83 ± 2.0 e	145 ± 4.8 g
T4	89 ± 1.4 gh	155 ± 1.8 i	74 ± 1.0 ef	129 ± 1.8 h
T5	205 ± 3.0 f	324 ± 10.3 g	177 ± 1.9 d	301 ± 2.8 f
T6	216 ± 2.1 e	344 ± 6.7f	179 ± 3.6 d	337 ± 12.1 d
T7	226 ± 2.8 d	412 ± 2.0 d	186 ± 2.3 d	357 ± 3.5 c
T8	224 ± 1.2 de	390 ± 1.3 e	179 ± 4.6 d	324 ± 5.0 e
T9	246 ± 1.0 c	426 ± 3.3 cd	201 ± 2.5 c	357 ± 8.2 c
T10	253 ± 2.7 bc	437 ± 2.0 bc	205 ± 8.1 bc	373 ± 5.4 b
T11	276 ± 4.6 a	474 ± 3.0 a	221 ± 1.0 a	434 ± 1.6 a
T12	258 ± 8.4 b	450 ± 4.6 b	211 ± 10.9 b	382 ± 6.7 b

* Means followed by the same letter are not significantly different by the protected LSD, P ≤ 0.05. Data are means value ± SE (n = 3). 1st cut and 2nd cut means the first and second cutts, respectively.

**Table 7:** The mean values of herb fresh weight (ton ha^−1^) of lovage plants as affected with plant spacing, melatonin concentrations and their combination treatments in two cuts of both seasons.

Treatments	First season		Second season	
	1^st^ cut	2^nd^ cut	1^st^ cut	2^nd^ cut
*Planting space between plants in row (cm)*				
15	9.5 ± 0.38 b*	16.1 ± 1.12 b	8.2 ± 0.32 b	14.2 ± 0.64 b
30	12.1 ± 0.21 a	20.4 ± 0.89 a	10.0 ± 0.11 a	18.3 ± 0.51 a
45	9.6 ± 0.19 b	16.5 ± 0.29 b	7.8 ± 0.15 c	14.3 ± 0.47 b

*Melatonin concentrations (µM)*				
Without soaking	9.7 ± 0.57 d	15.5 ± 1.02 d	8.2 ± 0.54 c	14.1 ± 0.88 c
0	10.1 ± 0.60 c	16.8 ± 0.78 c	8.5 ± 0.47 b	15.6 ± 1.01 b
75	11.3 ± 0.47 a	20.0 ± 0.98 a	9.2 ± 0.40 a	17.3 ± 0.79 a
100	10.6 ± 0.58 b	18.5 ± 1.00 b	8.7 ± 0.42 b	15.5 ± 0.79 b

*Interaction*				
T1	8.5 ± 0.85 f	12.6 ± 0.39 l	7.2 ± 0.44 g	12.2 ± 0.64 f
T2	9.0 ± 0.43 ef	15.0 ± 0.35 k	8.0 ± 0.18 de	14.3 ± 0.71 d
T3	10.5 ± 0.22 c	19.4 ± 0.09c	9.2 ± 0.22 c	16.1 ± 0.53 c
T4	9.9 ± 0.16 cd	17.2 ± 0.20 g	8.2 ± 0.11 d	14.3 ± 0.19 d
T5	11.4 ± 0.17 b	18.0 ± 1.29 e	9.8 ± 0.10 b	16.8 ± 0.15 c
T6	12.0 ± 0.13 ab	19.1 ± 0.37 d	9.9 ± 0.21 ab	18.7 ± 0.67 b
T7	12.6 ± 0.15 a	22.9 ± 0.12 a	10.4 ± 0.13 a	19.8 ± 0.19 a
T8	12.5 ± 0.06 a	21.7 ± 0.07 b	10.0 ± 0.27 ab	18.0 ± 0.27 b
T9	9.1 ± 0.04 ef	15.8 ± 0.12 j	7.4 ± 0.10 fg	13.2 ± 0.31 e
T10	9.4 ± 0.10 de	16.2 ± 0.09 i	7.6 ± 0.30 efg	13.8 ± 0.21 de
T11	10.2 ± 0.17 c	17.5 ± 0.12 f	8.2 ± 0.04 d	16.1 ± 0.06 c
T12	9.5 ± 0.31 de	16.7 ± 0.17 h	7.8 ± 0.41 def	14.2 ± 0.25 d

* Means followed by the same letter are not significantly different by the protected LSD, P ≤ 0.05. Data are means value ± SE (n = 3). 1st cut and 2nd cut means the first and second cutts, respectively.

### 3.4 Herb dry weight

Table 8, Table 9 shows the effect of different planting spaces and soaking treatments and their interactions on herb dry weight of lovage plants. The response varied according to the treatments. Table 8, Table 9 indicated that the 45 cm planting space gave the highest significant average of herb dry weight per plant (23.6 and 34.0 g plant^−1^ in the first season and 23.4 and 35.1 g plant^−1^ in the second season) in the two cuts across all melatonin concentrations. On the other hand, 30 cm planting space had the maximum herb dry weight per hectare (1052 and 1440 kg ha^−1^ in the first season and 1122 and 1726 kg ha^−1^ in the second season) for both cuts, respectively across all melatonin concentrations. Soaking seeds 75 µM melatonin solution recorded the maximum herb dry weight per plant (17.9 and 26.7 g plant^−1^ in the first season and 18.8 and 28.1 g plant^−1^ in the second season) as well as maximum herb dry weight per hectare (1005 and 1446 kg ha^−1^ in the first season and 1044 and 1560 kg ha^−1^ in the second one) across all planting space treatments in the first and second cuts, respectively. Using the treatment of 45 cm between plants in the same row plus 75 µM melatonin gave the maximum herb dry weight per plant (24.7 and 38.7 g plant^−1^ in the first season and 25.9 and 38.4 g plant^−1^ in the second season) compared with the other combination treatments in both cuts, respectively. From the other hand, the combination treatment of 30 cm between plants in the same row plus 75 µM melatonin recorded the heaviest herb dry weight per hectare (1127 and 1687 kg ha^−1^ in the first season and 1214 and 1852 kg ha^−1^ in the second season) for the first and second cuts, respectively.

**Table 8:** The mean values of herb dry weight (g plant^−1^) of lovage plants as affected with plant spacing, melatonin concentrations and their combination treatments in two cuts of both seasons.

Treatments	First season		Second season	
	1^st^ cut	2^nd^ cut	1^st^ cut	2^nd^ cut
*Planting space between plants in row (cm)*				
15	7.7 ± 0.8 c*	9.7 ± 1.6 c	7.6 ± 0.9 c	11.7 ± 1.4 c
30	18.9 ± 1.6 b	25.9 ± 3.6 b	20.2 ± 1.5 b	31.1 ± 2.9 b
45	23.6 ± 1.1 a	34.0 ± 3.8 a	23.4 ± 1.9 a	35.1 ± 3.2 a

*Melatonin concentrations (µM)*				
Without soaking	15.7 ± 6.8 c	20.4 ± 10.3 c	15.8 ± 6.6 c	22.6 ± 9.9 c
0	16.4 ± 7.1 bc	21.9 ± 10.2 bc	16.4 ± 6.9 c	25.6 ± 10.3 b
75	17.9 ± 7.2 a	26.7 ± 12.3 a	18.8 ± 7.8 a	28.1 ± 11.9 a
100	17.0 ± 7.4 ab	24.0 ± 10.6 b	17.3 ± 7.7 b	27.5 ± 11.4 a

*Interaction*				
T1	7.1 ± 0.3 e	7.5 ± 0.3 f	7.1 ± 0.9 f	9.8 ± 0.9 e
T2	7.5 ± 0.5 e	9.6 ± 0.5 f	7.5 ± 0.5 f	11.8 ± 0.3 de
T3	8.8 ± 0.4 e	11.0 ± 0.7 f	8.6 ± 0.7 f	12.6 ± 1.0 d
T4	7.5 ± 0.8 e	10.9 ± 1.2 f	7.2 ± 0.5 f	12.6 ± 0.9 d
T5	17.9 ± 0.3 d	23.7 ± 2.1 e	18.8 ± 0.4 e	26.7 ± 0.6 c
T6	18.4 ± 2.3 d	23.3 ± 3.1 e	19.3 ± 1.5 de	31.3 ± 0.6 b
T7	20.3 ± 1.5 c	30.4 ± 1.0 cd	21.9 ± 0.7 c	33.3 ± 1.5 b
T8	19.2 ± 1.2 cd	26.3 ± 3.2 de	20.9 ± 0.8 cd	32.9 ± 0.9 b
T9	22.2 ± 0.6 b	30.0 ± 4.4 cd	21.4 ± 0.8 c	31.3 ± 2.1 b
T10	23.4 ± 0.4 ab	32.7 ± 0.6 bc	22.4 ± 1.2 bc	33.5 ± 0.5 b
T11	24.7 ± 0.8 a	38.7 ± 1.2 a	25.9 ± 0.5 a	38.4 ± 1.6 a
T12	24.1 ± 0.4 a	34.7 ± 0.6 ab	23.9 ± 0.6 b	37.0 ± 1.1 a

* Means followed by the same letter are not significantly different by the protected LSD, P ≤ 0.05. Data are means value ± SE (n = 3). 1st cut and 2nd cut means the first and second cutts, respectively.

**Table 9:** The mean values of herb dry weight (kg ha^−1^) of lovage plants as affected with plant spacing, melatonin concentrations and their combination treatments in two cuts of both seasons.

Treatments	First season		Second season	
	1^st^ cut	2^nd^ cut	1^st^ cut	2^nd^ cut
*Planting space between plants in row (cm)*				
15	858 ± 37 b*	1082 ± 74 c	847 ± 40 b	1302 ± 65 b
30	1052 ± 37 a	1440 ± 84 a	1122 ± 36 a	1726 ± 67 a
45	875 ± 17 b	1259 ± 60 b	866 ± 29 b	1298 ± 49 b

*Melatonin concentrations (µM)*				
Without soaking	868 ± 41 c	1087 ± 97 c	875 ± 57 c	1244 ± 81 c
0	908 ± 46 bc	1191 ± 59 c	912 ± 55 bc	1433 ± 98 b
75	1005 ± 45 a	1446 ± 88 a	1044 ± 57 a	1560 ± 98 a
100	933 ± 49 b	1319 ± 67 b	950 ± 70 b	1532 ± 97 a

*Interaction*				
T1	786 ± 28 g	836 ± 31 f	789 ± 102 e	1090 ± 10 e
T2	836 ± 50 fg	1067 ± 53 e	836 ± 50 e	1316 ± 31 e
T3	972 ± 44 bcde	1219 ± 80 cde	960 ± 81 cd	1404 ± 111 cd
T4	838 ± 92 fg	1209 ± 134 de	803 ± 52 e	1397 ± 103 de
T5	994 ± 18 bcd	1315 ± 116 bcd	1042 ± 21 c	1481 ± 32 c
T6	1022 ± 126 abc	1296 ± 170 bcd	1069 ± 85 bc	1741 ± 32 bc
T7	1127 ± 81 a	1687 ± 56 a	1214 ± 37 a	1852 ± 85 a
T8	1065 ± 69 ab	1463 ± 179 b	1163 ± 47 ab	1830 ± 52 ab
T9	823 ± 24 fg	1111 ± 161 de	793 ± 31 e	1160 ± 77 e
T10	867 ± 16 efg	1210 ± 21de	831 ± 44 e	1242 ± 19 e
T11	915 ± 30 cdef	1432 ± 43 bc	957 ± 18 cd	1422 ± 61 cd
T12	894 ± 15defg	1284 ± 21 bcd	884 ± 22 de	1369 ± 39 e

* Means followed by the same letter are not significantly different by the protected LSD, P ≤ 0.05. Data are means value ± SE (n = 3). 1st cut and 2nd cut means the first and second cutts, respectively.

### 3.5 Root fresh and dry weights

Roots fresh and dry weights per plant and yield per hectare which were determined for lovage plants depending on studied treatments are shown in Table 10, Table 11. Results showed that plant spacing, seeds soaking and their combination treatments are significant factors that affecting roots fresh and dry weights per plant and per hectare in the first and second seasons. Cultivation of lovage seeds at 45 cm planting space had the maximum significant root fresh weight (135.5 and 143.8 g plant^−1^) and roots dry weight per plant (13.6 and 13.7 g plant^−1^) in both studied seasons compared with the other studied planting space. On the contrary, the maximum yield of roots fresh weight (7742 and 7565 kg ha^−1^) in both seasons were recorded with sowing seeds under 15 cm planting space conditions, while the maximum significant yield of roots dry weight per hectare (633 and 623 kg ha^−1^) in the first and second seasons were found with plants sown in 30 cm planting space compared with the other studied planting space. By comparing the melatonin concentrations, the application of 75 µM melatonin solution for soaking seeds of lovage was able to achieve the maximum significant roots fresh weight per plant (112.5 and 116.1 g plant^−1^) and per hectare (6709 and 6829 kg ha^−1^), roots dry weight per plant (10.9 and 10.6 g plant^−1^) and per hectare (624 and 621 kg ha^−1^) in both studied seasons, respectively compared with the other studied soaking period of seeds. For the combination treatments of planting spaces and melatonin concentrations, the eleven combination treatment of 45 cm between plants in row plus using 75 µM melatonin solution before sowing recoded the maximum roots fresh weight (139.4 and 149.5 g plant^−1^) and roots dry weight per plant (14.6 and 14.0 g plant^−1^) in both studied seasons, respectively. However, the maximum yield of roots fresh weight (7927 and 7808 kg ha^−1^) in both seasons and roots dry weight per hectare (702 kg ha^−1^) in the second one were recorded with the third combination treatment of 15 cm between plants in row plus soaking seeds in 75 µM melatonin solution before sowing seeds but the maximum yield of roots dry weight per hectare (669 kg ha^−1^) in the first season was noticed with plants of the seventh combination treatment of 30 cm between plants in row plus using 75 µM melatonin before the sowing compared with the other studied combination treatments.

**Table 10:** The mean values of **roots fresh weight (g plant^−1^)** and yield of roots fresh weight (kg ha^−1^) of lovage plants as affected with plant spacing, melatonin concentrations and their combination treatments in two cuts of both seasons.

Treatments	Roots fresh weight (g plant^−1^)		Roots fresh weight (kg ha^−1^)	
	First season	Second season	First season	Second season
*Planting space between plants in row (cm)*				
15	69.7 ± 1.5 c*	68.1 ± 2.0 c	7742 ± 71 a	7565 ± 94 a
30	124.8 ± 2.2 b	126.2 ± 2.1 b	6933 ± 51 b	7009 ± 49 b
45	135.5 ± 2.8 a	143.8 ± 5.1 a	5019 ± 43 c	5326 ± 80 c

*Melatonin concentrations (µM)*				
Without soaking	107.7 ± 30.3 c	109.4 ± 33.5 d	6418 ± 491c	6438 ± 420 d
0	109.6 ± 30.4 b	111.2 ± 33.2 c	6548 ± 515 b	6569 ± 446 c
75	112.5 ± 31.3 a	116.1 ± 35.6 a	6709 ± 514 a	6829 ± 426 a
100	110.2 ± 30.5 b	114.0 ± 35.2 b	6584 ± 517 b	6697 ± 413 b

*Interaction*				
T1	67.8 ± 1.0 f	65.5 ± 1.1 f	7533 ± 110 c	7280 ± 125 c
T2	69.5 ± 0.9 ef	67.9 ± 0.4 ef	7725 ± 97b	7539 ± 48 b
T3	71.3 ± 1.1 e	70.3 ± 1.0 e	7927 ± 126 a	7808 ± 113 a
T4	70.1 ± 0.2 ef	68.7 ± 1.4 e	7783 ± 21 ab	7633 ± 157 ab
T5	122.4 ± 2.4 d	124.3 ± 0.7 d	6800 ± 131 e	6906 ± 39 e
T6	125.0 ± 1.4 cd	125.5 ± 1.7 d	6943 ± 75 de	6974 ± 97 de
T7	126.7 ± 1.9 c	128.6 ± 0.5 c	7037 ± 107 d	7144 ± 29 cd
T8	125.2 ± 0.8 cd	126.3 ± 2.4 cd	6954 ± 42 de	7014 ± 133 de
T9	132.9 ± 0.7 b	138.4 ± 1.8 b	4921 ± 25 g	5127 ± 68 g
T10	134.4 ± 1.8 b	140.3 ± 2.2 b	4978 ± 68 g	5195 ± 83 g
T11	139.4 ± 0.6 a	149.5 ± 2.5 a	5162 ± 24 f	5536 ± 91f
T12	135.4 ± 2.0 b	147.0 ± 2.1 a	5014 ± 73 fg	5444 ± 78f

* Means followed by the same letter are not significantly different by the protected LSD, P ≤ 0.05. Data are means value ± SE (n = 3). 1st cut and 2nd cut means the first and second cutts, respectively.

**Table 11:** The mean values of roots dry weight (g plant^−1^) and roots dry weight (kg ha^−1^) of lovage plants as affected with plant spacing, melatonin concentrations and their combination treatments in two cuts of both seasons.

Treatments	Roots dr y weight (g plant^−1^)		Roots dry weight (kg ha^−1^)	
	First season	Second season	First season	Second season
*Planting space between plants in row (cm)*				
15	5.3 ± 0.5 c*	5.5 ± 1.1 c	583 ± 61 b	589 ± 76 b
30	11.4 ± 0.7 b	11.2 ± 0.6 b	633 ± 40 a	623 ± 32 a
45	13.6 ± 0.7 a	13.7 ± 0.4 a	503 ± 28 c	506 ± 16 c

*Melatonin concentrations (µM)*				
Without soaking	9.4 ± 3.7 d	9.5 ± 2.4 c	526 ± 55 d	535 ± 48 c
0	9.8 ± 3.7 c	9.9 ± 2.5 bc	558 ± 56 c	555 ± 58 bc
75	10.9 ± 3.9 a	10.6 ± 1.6 a	624 ± 63 a	621 ± 83 a
100	10.3 ± 3.8 b	10.2 ± 2.1 b	584 ± 71 b	580 ± 58 b

*Interaction*				
T1	4.6 ± 0.2 h	4.7 ± 0.3 e	514 ± 26 fg	522 ± 28 d
T2	5.1 ± 0.2 h	4.8 ± 0.4 e	564 ± 20 de	538 ± 4 5 d
T3	6.0 ± 0.2 g	6.3 ± 0.5 d	661 ± 24 ab	702 ± 50 a
T4	5.3 ± 0.4 gh	5.4 ± 0.2 e	593 ± 41 cd	596 ± 23 c
T5	10.6 ± 0.5 f	10.6 ± 0.7 c	591 ± 27 cd	589 ± 38 c
T6	11.1 ± 0.4 ef	11.2 ± 0.6 bc	617 ± 20 bc	624 ± 32 bc
T7	12.0 ± 0.3 d	11.6 ± 0.3 b	669 ± 14 a	643 ± 14 b
T8	11.8 ± 0.7 de	11.4 ± 0.4 b	656 ± 39 ab	635 ± 22 bc
T9	12.8 ± 0.3 c	13.3 ± 0.0 a	473 ± 13 g	493 ± 0 d
T10	13.3 ± 0.3 bc	13.6 ± 0.4 a	493 ± 13 g	505 ± 15 d
T11	14.6 ± 0.3 a	14.0 ± 0.6 a	542 ± 10 ef	519 ± 23 d
T12	13.6 ± 0.2 b	13.7 ± 0.2 a	504 ± 8 fg	509 ± 8 d

* Means followed by the same letter are not significantly different by the protected LSD, P ≤ 0.05. Data are means value ± SE (n = 3). 1st cut and 2nd cut means the first and second cutts, respectively.

### 3.6 Chlorophyll, total phenolic and antioxidant content

The results of chlorophyll content, total phenolic and antioxidant content means comparison of lovage plants as affected with planting spacing, melatonin concentrations and their combination treatments are shown in Table 12, Table 13, Table 14. The highest means values of chlorophyll content (49.69 and 52.49 spad unit in the first season and 51.58 and 48.40 spad unit in the second season) for both cuts, respectively were noticed with planting distance of 45 cm between plants in the same row with insignificant differences between the means values in the second cut of the second season. While, The maximum means values of total phenolic (14.28 and 13.54 mg gallic 1 g^−1^ herb in the first season and 14.25 and 13.90 mg gallic 1 g^−1^ herb in the second season) and antioxidant content (53.94 and 54.49 mg TE 100 g^−1^ herb in the first season and 56.63 and 55.57 mg TE 100 g^−1^ herb in the second season) for studied cuts, respectively was observed in lovage plants sown in 15 cm planting spaces conditions compared with the other different studied planting spaces in both seasons, respectively. Based on melatonin application, soaking lovage seeds before sowing 75 µM melatonin solution gave the highest significant means values of chlorophyll content (48.88 and 49.37 spad unit in the first cut and 50.61 spad unit in the second cut), total phenolic (12.98 and 12.80 mg gallic 1 g^−1^ herb in the first cut and 12.39 and 12.59 mg gallic 1 g^−1^ herb in the second cut) and antioxidant content (50.11 and 51.66 mg TE 100 g^−1^ herb in the first cut and 49.57 and 50.57 mg TE 100 g^−1^ herb in the second cut) in both seasons except the maximum significant means value of chlorophyll content (52.06 spad unit) in the second cut of the second season were found with melatonin concentration of 100 µM compared with the other studied concentrations. Regarding the effect of combination treatments, the combination treatment of 45 cm between plants in row plus 75 µM melatonin solution gave the maximum values of chlorophyll content (52.00 and 56.27 spad unit) and (52.37 and 55.03 spad unit) in both cuts of the two seasons. Moreover, the combination treatment of T3 (15 cm between plants in row and 75 µM melatonin) had the maximum mean values of total phenolic (14.96 and 14.10 mg gallic 1 g^−1^ herb in the first season and 14.62 and 14.27 mg gallic 1 g^−1^ herb in the second season) and antioxidant content (54.32 and 55.33 mg TE 100 g^−1^ herb in the first season and 57.79 and 56.95 mg TE 100 g^−1^ herb in the second season) for the first and the second cuts, respectively.

**Table 12:** The mean values of chlorophyll content (SPAD unit) of lovage plants as affected with planting spacing, melatonin concentrations and their combination treatments in two cuts of both seasons.

Treatments	First season		Second season	
	1^st^ cut	2^nd^ cut	1^st^ cut	2^nd^ cut
*Planting space between plants in row (cm)*				
15	44.90 ± 2.81 b*	42.83 ± 2.32 c	42.17 ± 2.36 c	46.48 ± 4.04 a
30	45.98 ± 1.69 b	48.30 ± 1.92 b	49.34 ± 1.85 b	47.27 ± 4.91 a
45	49.69 ± 2.46 a	52.49 ± 2.58 a	51.58 ± 1.43 a	48.40 ± 3.43 a

*Melatonin concentrations (µM)*				
Without soaking	44.42 ± 3.03 c	45.99 ± 5.03 c	45.70 ± 5.22 c	44.40 ± 3.79 c
0	46.00 ± 2.20 b	47.73 ± 4.16 b	47.70 ± 3.88 b	45.49 ± 2.99 c
75	48.88 ± 2.59 a	50.61 ± 4.85 a	49.37 ± 3.44 a	48.92 ± 1.81 b
100	48.13 ± 2.72 a	47.16 ± 3.61 bc	48.01 ± 5.06 b	52.06 ± 2.81 a

*Interaction*				
T1	41.43 ± 1.00 e	39.80 ± 1.45 h	39.27 ± 0.68 g	41.67 ± 0.47 d
T2	43.83 ± 1.85 de	42.50 ± 1.75 g	42.83 ± 0.32 ef	44.90 ± 4.09 cd
T3	47.40 ± 0.60 c	45.23 ± 0.38 ef	45.07 ± 0.81 de	49.43 ± 1.76 b
T4	46.93 ± 1.85 cd	43.77 ± 0.68 fg	41.50 ± 1.68 fg	49.93 ± 0.59 b
T5	43.97 ± 1.85 de	47.47 ± 1.24 de	47.10 ± 1.71 cd	42.13 ± 0.76 d
T6	46.07 ± 1.32 cd	49.40 ± 0.66 cd	49.20 ± 0.56 bc	48.03 ± 1.45 bc
T7	47.23 ± 0.70 c	50.33 ± 0.72 bc	50.67 ± 1.53 ab	48.40 ± 1.97 bc
T8	46.67 ± 0.95 cd	46.00 ± 0.90 ef	50.40 ± 1.22 ab	48.93 ± 2.30 b
T9	47.87 ± 0.92 bc	50.70 ± 1.91 bc	50.73 ± 1.72 ab	49.40 ± 0.44 b
T10	48.10 ± 0.70 bc	51.30 ± 1.11 bc	51.07 ± 2.00 ab	43.53 ± 1.01 d
T11	52.00 ± 2.00 a	56.27 ± 1.42 a	52.37 ± 0.91 a	55.03 ± 0.84 a
T12	50.80 ± 3.04 ab	51.70 ± 0.72 b	52.13 ± 0.76 a	51.2 ± 3.06 b

* Means followed by the same letter are not significantly different by the protected LSD, P ≤ 0.05. Data are means value ± SE (n = 3). 1st cut and 2nd cut means the first and second cutts, respectively.

**Table 13:** The mean values of total phenolic (mg gallic 1 g^−1^ herb) of lovage plants as affected with planting spacing, melatonin concentrations and their combination treatments in two cuts of both seasons.

Treatments	First season		Second season	
	1^st^ cut	2^nd^ cut	1^st^ cut	2^nd^ cut
*Planting space between plants in row (cm)*				
15	14.28 ± 0.55 a*	13.54 ± 0.59 a	14.25 ± 0.51 a	113.90 ± 0.35 a
30	12.18 ± 0.64 b	11.62 ± 0.55b	12.20 ± 0.58 b	111.94 ± 0.63 b
45	10.20 ± 0.73 c	9.62 ± 0.72c	110.19 ± 0.66 c	110.00 ± 0.71 c

*Melatonin concentrations (µM)*				
Without soaking	11.54 ± 1.92 d	10.86 ± 1.76 d	11.67 ± 1.85 b	11.31 ± 1.89 d
0	11.99 ± 1.91 c	11.44 ± 1.72c	11.85 ± 1.92 b	11.76 ± 1.88 c
75	12.98 ± 1.69 a	12.39 ± 1.52 a	12.80 ± 1.58 a	12.59 ± 1.46 a
100	12.37 ± 1.66 b	11.68 ± 1.84b	12.52 ± 1.79 a	12.13 ± 1.63 b

*Interaction*				
T1	13.80 ± 0.12 b	12.73 ± 0.32c	13.81 ± 0.37 b	13.53 ± 0.12 b
T2	14.11 ± 0.06 b	13.43 ± 0.21b	14.04 ± 0.21 ab	13.83 ± 0.06 ab
T3	14.96 ± 0.62 a	14.10 ± 0.26 a	14.62 ± 0.68 a	14.27 ± 0.29 a
T4	14.25 ± 0.43 b	13.90 ± 0.10 a	14.52 ± 0.36 a	13.97 ± 0.42 ab
T5	11.40 ± 0.34 e	11.12 ± 0.08 d	11.61 ± 0.29 c	11.17 ± 0.33 e
T6	12.12 ± 0.50 d	11.42 ± 0.21 d	11.84 ± 0.55 d	11.88 ± 0.49 d
T7	12.82 ± 0.26 c	12.47 ± 0.15c	12.71 ± 0.33 c	12.57 ± 0.25 c
T8	12.38 ± 0.48 cd	11.47 ± 0.15 d	12.62 ± 0.12 c	12.13 ± 0.47 cd
T9	9.40 ± 0.37 g	8.72 ± 0.12 g	9.58 ± 0.09 g	9.22 ± 0.36 g
T10	9.74 ± 0.20 g	9.48 ± 0.12 f	9.67 ± 0.25 g	9.55 ± 0.19 g
T11	11.15 ± 0.05 ef	10.61 ± 0.16 e	11.09 ± 0.17 e	10.93 ± 0.05 e
T12	10.49 ± 0.09 f	9.68 ± 0.25 f	10.41 ± 0.16f	10.29 ± 0.09 f

* Means followed by the same letter are not significantly different by the protected LSD, P ≤ 0.05. Data are means value ± SE (n = 3). 1st cut and 2nd cut means the first and second cutts, respectively.

**Table 14:** The mean values of antioxidant content (mg TE 100 g^−1^ herb) of lovage plants as affected with planting spacing, melatonin concentrations and their combination treatments in two cuts of both seasons.

Treatments	First season		Second season	
	1^st^ cut	2^nd^ cut	1^st^ cut	2^nd^ cut
*Planting space between plants in row (cm)*				
15	53.94 ± 0.82 a*	54.49 ± 1.52 a	56.63 ± 1.18 a	55.57 ± 1.49 a
30	48.34 ± 2.27 b	47.83 ± 2.29 b	49.61 ± 1.65 b	48.24 ± 2.62 b
45	40.02 ± 3.00 c	39.96 ± 2.58 c	41.48 ± 3.24 c	40.32 ± 2.48 c

*Melatonin concentrations (µM)*				
Without soaking	45.42 ± 7.61 c	45.37 ± 6.72 b	47.20 ± 7.97 d	45.46 ± 7.12 d
0	46.94 ± 6.31 b	46.36 ± 7.38 b	48. 17 ± 7.29 c	47.30 ± 7.31 c
75	50.11 ± 4.65 a	49.57 ± 5.20 a	51. 66 ± 5.58 a	50.57 ± 5.75 a
100	47.26 ± 5.93 b	48.41 ± 6.27 a	49. 94 ± 5.61 b	48.84 ± 6.50 b

*Interaction*				
T1	53.73 ± 0.83 a	52.97 ± 1.34 a	55.65 ± 0.60 b	54.00 ± 0.40 b
T2	53.88 ± 0.91 a	54.73 ± 0.31 a	56.37 ± 1.72 ab	55.47 ± 0.23 ab
T3	54.32 ± 1.06 a	55.33 ± 0.83 a	57.79 ± 0.51 a	56.95 ± 1.60 a
T4	53.82 ± 0.87 a	54.93 ± 2.27 a	56.72 ± 0.73 ab	55.87 ± 1.67 a
T5	46.26 ± 0.33 c	45.55 ± 1.12 cd	48.55 ± 0.48 d	44.69 ± 1.33 e
T6	47.51 ± 0.89 c	46.44 ± 2.15 c	48.47 ± 0.48 d	47.73 ± 1.66 d
T7	51.86 ± 0.64 b	49.87 ± 1.29 b	52.20 ± 0.52 c	50.93 ± 0.46 c
T8	47.70 ± 0.64 c	49.47 ± 0.46 b	49.23 ± 0.49 d	49.60 ± 0.40 c
T9	36.25 ± 0.48 f	37.59 ± 0.35 f	37.41 ± 0.38 g	37.70 ± 0.31 g
T10	39.42 ± 0.48 e	37.90 ± 0.98 f	39.67 ± 0.40 f	38.71 ± 0.35 g
T11	44.15 ± 0.69 d	43.50 ± 0.66 d	44.98 ± 0.72 e	43.82 ± 0.18 e
T12	40.26 ± 1.02 e	40.84 ± 0.63 e	43.86 ± 0.44 e	41.04 ± 0.30 f

* Means followed by the same letter are not significantly different by the protected LSD, P ≤ 0.05. Data are means value ± SE (n = 3). 1st cut and 2nd cut means the first and second cutts, respectively.

### 3.7 Essential oil content

Table 15, Table 16, Table 17 shows the ability of plant spacing, soaking seeds and their interaction to induce significant differences in the essential oil percentage, essential oil per plant and per hectare in the first and second seasons and in both cuts. Despite the highest essential oil percentage of lovage plants (0.568 and 0.455% in the first season and 0.569 and 0.494% in the second season) that recorded with using the closer spacing (15 cm), the maximum essential oil per plant (0.101 mL plant^−1^ in the first season) in the first cut and (0.113 and 0.142 mL plant^−1^ in the second season) in both cuts and also, per hectare (5.61 and 7.52 L ha^−1^ in the first season and 6.30 and 7.87 L ha^−1^ in the second season) for the first and second cuts, respectively were found with using medium space of 30 cm. Both of 30 and 45 cm between plants had the maximum significant essential oil content per plant in the second cut of the first season. By comparing the used melatonin concentrations, the application of 75 µM melatonin concentration gave the maximum essential oil percentage, essential oil content per plant and per hectare (0.559 and 0.447%, 0.095 and 0.115 mL plant^−1^ and 5.66 and 6.50 L ha^−1^) in the first season and (0.597 and 0.503%, 0.106 and 0.134 mL plant^−1^ and 6.30 and 7.84 L ha^−1^) in the second season for both cuts, respectively compared with the other melatonin concentrations. Insignificant differences were detected between 0 and 75 µM melatonin concentrations in the second cut of the first season for essential oil yield per hectare. Furthermore, the results obtained indicate that the greatest essential oil percent (0.629 and 0.495% in the first season and 0.687 and 0.578% in the second season) for both cuts, respectively were obtained with combination treatment of plant spacing 15 cm plus 75 µM melatonin. While, the maximum essential oil content per plant (0.124 mL plant^−1^ in the first cut of the first season and 0.140 and 0.172 mL plant^−1^ in both cuts of the second season) and the greatest essential oil yield per hectare (6.87 and 7.56 L ha^−1^ in the first season and 7.76 and 9.57 L ha^−1^ in the second season) for both cuts, respectively were obtained from the combination treatment of 30 cm between plants in row and 75 µM melatonin, the maximum essential oil content per plant (0.154 mL plant^−1^) in the second cut of the first season was found with the combination treatment of 45 cm between plants and 75 µM melatonin concentration before planting.

**Table 15:** The mean values of essential oil percentage (%) of lovage plants as affected with plant spacing, melatonin concentrations and their combination treatments in two cuts of both seasons.

Treatments	First season		Second season	
	1^st^ cut	2^nd^ cut	1^st^ cut	2^nd^ cut
*Planting space between plants in row (cm)*				
15	0.568 ± 0.061 a*	0.455 ± 0.064 a	0.569 ± 0.103 a	0.494 ± 0.073 a
30	0.530 ± 0.070 b	0.413 ± 0.043 b	0.558 ± 0.067 a	0.450 ± 0.07 2b
45	0.374 ± 0.042 c	0.322 ± 0.051 c	0.402 ± 0.039 b	0.352 ± 0.05 2c

*Melatonin concentrations (µM)*				
Without soaking	0.425 ± 0.064 c	0.330 ± 0.051 d	0.419 ± 0.042 c	0.341 ± 0.053 d
0	0.489 ± 0.113 b	0.386 ± 0.061 c	0.509 ± 0.097 b	0.430 ± 0.07 4c
75	0.559 ± 0.096 a	0.447 ± 0.049 a	0.597 ± 0.104 a	0.503 ± 0.080 a
100	0.490 ± 0.102 b	0.425 ± 0.090 b	0.515 ± 0.095 b	0.455 ± 0.06 0b

*Interaction*				
T1	0.488 ± 0.007 e	0.372 ± 0.016 cd	0.415 ± 0.006 f	0.401 ± 0.031 d
T2	0.594 ± 0.038 abc	0.441 ± 0.018 b	0.584 ± 0.011 cd	0.484 ± 0.011 bc
T3	0.629 ± 0.020 a	0.495 ± 0.040 a	0.687 ± 0.013 a	0.578 ± 0.063 a
T4	0.563 ± 0.053 bcd	0.513 ± 0.042 a	0.591 ± 0.010 c	0.510 ± 0.017 bc
T5	0.437 ± 0.028 f	0.353 ± 0.015 de	0.467 ± 0.018 e	0.336 ± 0.006 e
T6	0.524 ± 0.034 de	0.409 ± 0.017 bc	0.562 ± 0.022 d	0.474 ± 0.01 1c
T7	0.611 ± 0.040 ab	0.448 ± 0.022 b	0.639 ± 0.036 b	0.517 ± 0.01 3b
T8	0.549 ± 0.013 cd	0.444 ± 0.190 b	0.565 ± 0.023 cd	0.474 ± 0.01 1c
T9	0.349 ± 0.022 g	0.265 ± 0.011 g	0.374 ± 0.015 g	0.284 ± 0.00 7f
T10	0.349 ± 0.022 g	0.309 ± 0.013 f	0.382 ± 0.020 g	0.332 ± 0.006 e
T11	0.437 ± 0.280 f	0.397 ± 0.016 c	0.464 ± 0.018 e	0.413 ± 0.027 d
T12	0.359 ± 0.007 g	0.316 ± 0.016 ef	0.390 ± 0.003 fg	0.379 ± 0.009 d

* Means followed by the same letter are not significantly different by the protected LSD, P ≤ 0.05. Data are means value ± SE (n = 3). 1st cut and 2nd cut means the first and second cutts, respectively.

**Table 16:** The mean values of essential oil content (mL plant^−1^) of lovage plants as affected with plant spacing, melatonin concentrations and their combination treatments in two cuts of both seasons. Data are means value ± SE (n = 3).

Treatments	First season		Second season	
	1^st^ cut	2^nd^ cut	1^st^ cut	2^nd^ cut
*Planting space between plants in row (cm)*				
15	0.044 ± 0.008 c*	0.044 ± 0.012 b	0.044 ± 0.011 c	0.058 ± 0.013 c
30	0.101 ± 0.019 a	0.110 ± 0.022 a	0.113 ± 0.020 a	0.142 ± 0.032 a
45	0.089 ± 0.013 b	0.111 ± 0.029 a	0.095 ± 0.017 b	0.125 ± 0.028 b

*Melatonin concentrations (µM)*				
Without soaking	0.063 ± 0.022 c	0.064 ± 0.028 d	0.066 ± 0.027 d	0.073 ± 0.025 d
0	0.075 ± 0.074 b	0.081 ± 0.032 c	0.079 ± 0.029 c	0.106 ± 0.040 c
75	0.095 ± 0.096 a	0.115 ± 0.046 a	0.106 ± 0.037 a	0.134 ± 0.047 a
100	0.078 ± 0.078 b	0.094 ± 0.028 b	0.084 ± 0.033 b	0.120 ± 0.047 b

*Interaction*				
T1	0.035 ± 0.002 e	0.028 ± 0.002 h	0.030 ± 0.004 h	0.040 ± 0.007
T2	0.045 ± 0.005 de	0.039 ± 0.002 gh	0.044 ± 0.002 g	0.057 ± 0.001 h
T3	0.055 ± 0.004 cde	0.054 ± 0.003 fg	0.059 ± 0.004f	0.073 ± 0.005 g
T4	0.042 ± 0.003 e	0.056 ± 0.008 f	0.043 ± 0.003 g	0.064 ± 0.003 gh
T5	0.078 ± 0.005 bcd	0.083 ± 0.007 e	0.088 ± 0.004 de	0.090 ± 0.002 f
T6	0.097 ± 0.018 ab	0.103 ± 0.009 cd	0.108 ± 0.012c	0.149 ± 0.006 cd
T7	0.124 ± 0.003 a	0.136 ± 0.008b	0.140 ± 0.007 a	0.172 ± 0.006 a
T8	0.105 ± 0.006 ab	0.117 ± 0.018 c	0.118 ± 0.001 bc	0.156 ± 0.006 bc
T9	0.078 ± 0.007 bcd	0.079 ± 0.012 e	0.080 ± 0.002 e	0.089 ± 0.008 f
T10	0.082 ± 0.007 bc	0.101 ± 0.006 d	0.086 ± 0.009 de	0.111 ± 0.001 e
T11	0.108 ± 0.009 ab	0.154 ± 0.010 a	0.120 ± 0.007 b	0.158 ± 0.004 b
T12	0.087 ± 0.003 bc	0.110 ± 0.004 cd	0.093 ± 0.002 d	0.140 ± 0.001 d

* Means followed by the same letter are not significantly different by the protected LSD, P ≤ 0.05. Data are means value ± SE (n = 3). 1st cut and 2nd cut means the first and second cutts, respectively.

**Table 17:** The mean values of yield of essential oil (l ha^−1^) of lovage plants as affected with plant spacing, melatonin concentrations and their combination treatments in two cuts of both seasons.

Treatments	First season		Second season	
	1^st^ cut	2^nd^ cut	1^st^ cut	2^nd^ cut
*Planting space between plants in row (cm)*				
15	4.91 ± 0.39 b*	4.92 ± 0.58 b	4.87 ± 0.53 b	6.49 ± 0.62 b
30	5.61 ± 0.45 a	7.52 ± 1.12 a	6.30 ± 0.48 a	7.87 ± 0.77 a
45	3.28 ± 0.21 c	4.11 ± 0.45 c	3.51 ± 0.26 c	4.62 ± 0.43 c

*Melatonin concentrations (µM)*				
Without soaking	3.69 ± 0.29 c	3.56 ± 0.37 c	3.70 ± 0.39 c	4.22 ± 0.35 d
0	4.46 ± 0.52 b	6.42 ± 1.56 a	4.69 ± 0.54 b	6.25 ± 0.76 c
75	5.66 ± 0.55 a	6.50 ± 0.39 a	6.26 ± 0.63 a	7.84 ± 0.69 a
100	4.58 ± 0.49 b	5.59 ± 0.56 b	4.92 ± 0.57 b	6.99 ± 0.64 b
Interaction				
T1	3.84 ± 0.19 fg	3.11 ± 0.20 fg	3.28 ± 0.46 d	4.40 ± 0.75 gh
T2	4.97 ± 0.61 de	4.36 ± 0.20 e	4.88 ± 0.20 c	6.37 ± 0.10 d
T3	6.12 ± 0.42 b	6.02 ± 0.27 c	6.59 ± 0.46 b	8.08 ± 0.51 b
T4	4.69 ± 0.32 e	6.21 ± 0.85 c	4.74 ± 0.35 c	7.12 ± 0.30 c
T5	4.34 ± 0.32 ef	4.64 ± 0.41 de	4.87 ± 0.21 c	4.97 ± 0.11 fg
T6	5.38 ± 0.98 cd	11.36 ± 1.06 a	6.02 ± 0.67 b	8.25 ± 0.34 b
T7	6.87 ± 0.20 a	7.56 ± 0.44 b	7.76 ± 0.38 a	9.57 ± 0.36 a
T8	5.84 ± 0.35 bc	6.51 ± 0.99 bc	6.56 ± 0.05 b	8.67 ± 0.34 b
T9	2.88 ± 0.25 h	2.94 ± 0.45 g	2.96 ± 0.06 d	3.30 ± 0.29 i
T10	3.03 ± 0.24 h	3.74 ± 0.21 efg	3.18 ± 0.33 d	4.13 ± 0.04 h
T11	3.99 ± 0.32 f	5.69 ± 0.39 cd	4.44 ± 0.25 c	5.86 ± 0.15 de
T12	3.21 ± 0.12 gh	4.06 ± 0.16 ef	3.45 ± 0.06 d	5.19 ± 0.03 ef

* Means followed by the same letter are not significantly different by the protected LSD, P ≤ 0.05. Data are means value ± SE (n = 3). 1st cut and 2nd cut means the first and second cutts, respectively.

### 3.8 Essential oil constituents

**Fig. 1:**
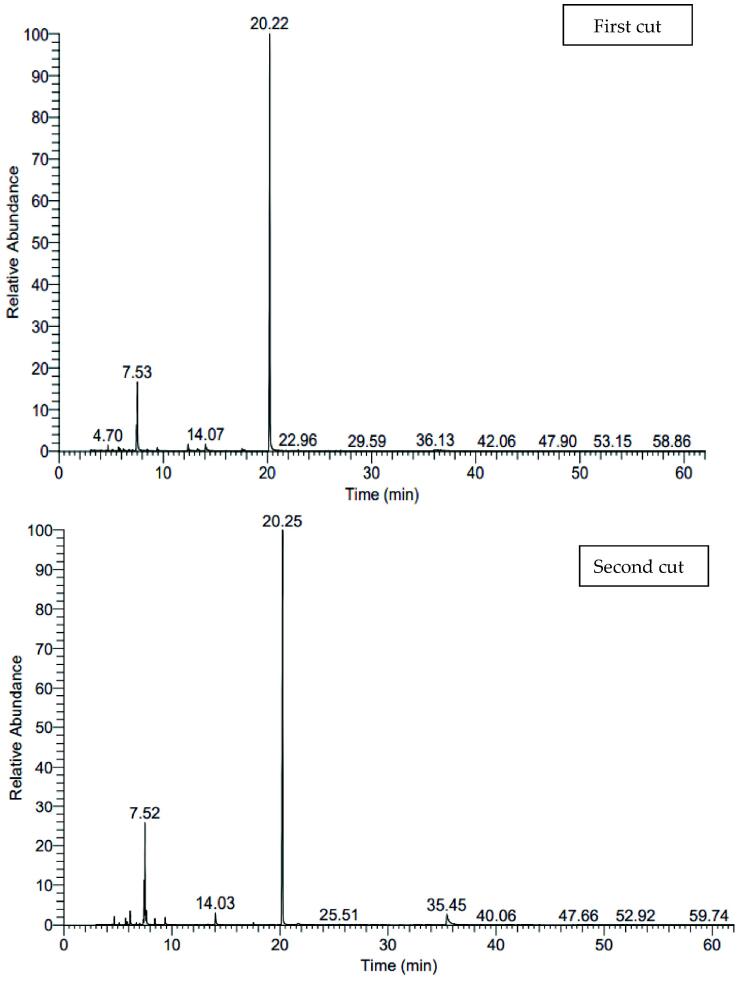
The pictogram of GC–MS for 15 cm planting space plus without soaking treatment in both cuts.

**Fig. 2:**
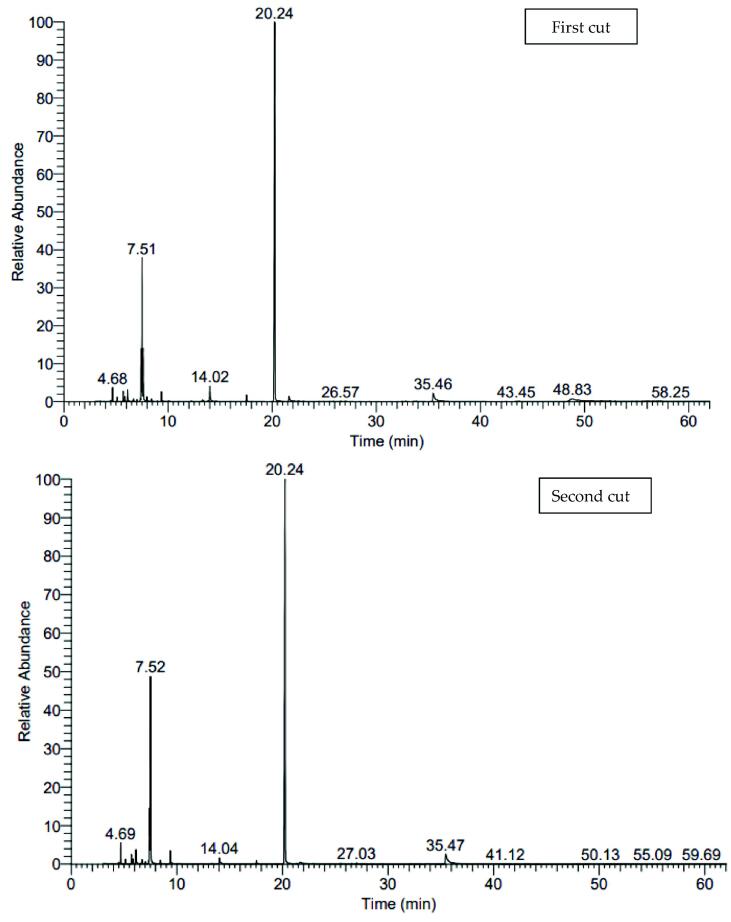
The pictogram of GC–MS for 30 cm planting space plus 75 µM melatonin treatment in both cuts.

**Fig. 3:**
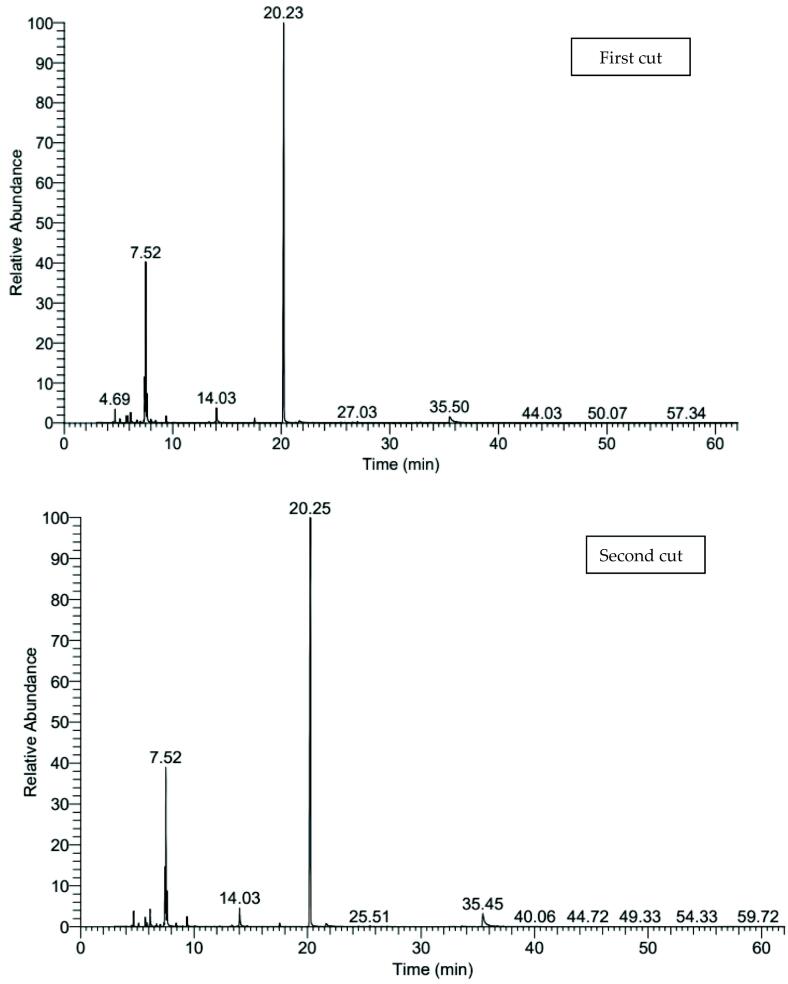
The pictogram of GC–MS for 30 cm planting space plus 100 µM melatonin treatment in both cuts.

**Fig. 4:**
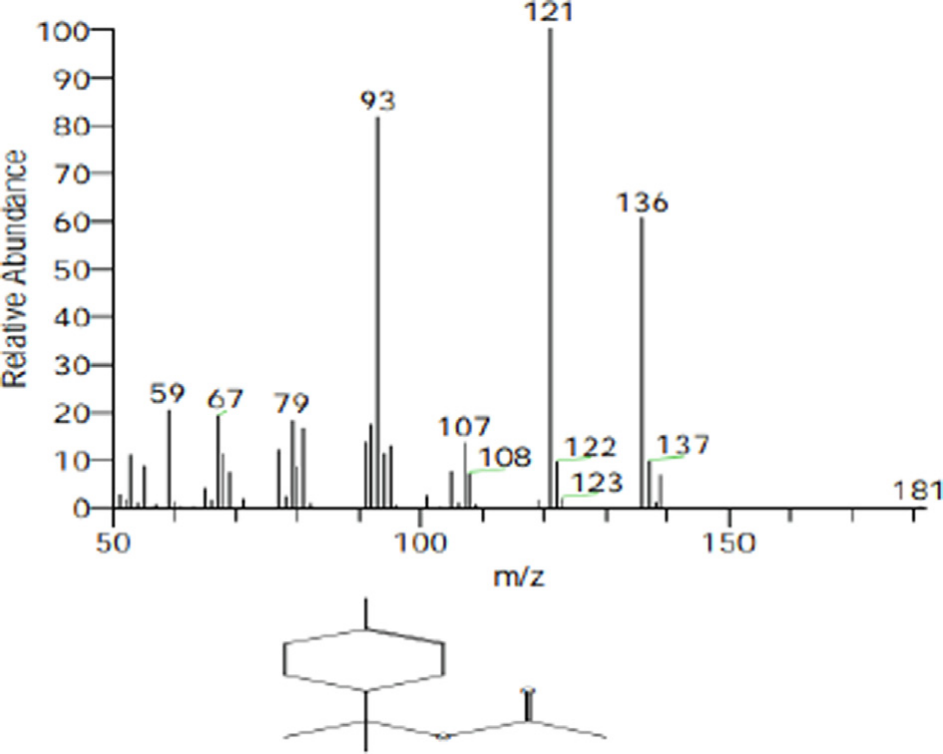
Structure of α-terpinyle acetate present in herb essential oil of lovage by using GC–MS analysis.

Fig. 1, Fig. 2, Fig. 3 and Table 18 shows the variable changes in the relative percentages of different ingredients of the essential oil distilled from the herb of lovage. It can be remarked that about 37 compounds were identified from essential oil of herb of the first and second cuts that represented 99.91–100% of lovage essential oil. The total oxygenated compounds that were ranged from 66.87 to 82.68% were superior comparing with hydrocarbon compounds which were ranged from 17.32 to 33.04 under all studied combination treatments in both cuts. The first major compound is α-terpinyle acetate (62.79:77.72%) followed by β-Phellandrene (10.24:19.18%). The maximum relative pronounce of α-terpinyle acetate (Fig. 4) were obtained with the combination treatment of 15 cm plant spacing and 0 µM melatonin in the first cut while the maximum relative pronounce of β-Phellandrene (19.18%) was recorded with the combination treatment of 30 cm plant spacing and seeds soaking solution of 75 µM melatonin in the second cut.

**Table 18:** Chemical analysis of lovage herb essential oil as affected with planting spacing and melatonin concentrations combination treatments in the second season.

Essential oil compounds		Molecular formula	RT	Relative percentage of the main constituents					
				15 cm planting space plus without soaking		30 cm planting space plus 75 µM melatonin		30 cm planting space plus 100 µM melatonin	
				1st cut	2nd cut	1st cut	2nd cut	1st cut	2nd cut
1	α-Thujene	C_10_H_16_	4.49	0.00	0.11	0.12	0.16	0.00	0.13
2	α-Pinene (-)-	C_10_H_16_	4.69	0.57	0.74	1.11	1.70	1.13	1.09
3	Camphene	C_10_H_16_	5.13	0.18	0.20	0.37	0.42	0.37	0.27
4	Sabinene	C_10_H_16_	5.71	0.53	0.68	0.93	0.88	0.67	0.76
5	2-α-Pinene	C_10_H_16_	5.87	0.27	0.31	0.46	0.46	0.69	0.30
6	α-Myrcene	C_10_H_16_	6.13	0.44	1.71	1.22	1.64	1.25	1.82
7	α -Phellandrene	C_10_H_16_	6.71	0.22	0.27	0.29	0.46	0.30	0.29
8	α-Terpinene	C_10_H_16_	7.03	0.16	0.20	0.21	0.23	0.17	0.21
9	o-Cymene	C_10_H_14_	7.34	0.00	0.30	0.16	0.18	0.17	0.16
10	(+)-carvene	C_10_H_16_	7.43	3.33	4.44	4.81	5.45	4.37	5.09
11	β-Phellandrene	C_10_H_16_	7.52	10.24	10.69	13.28	19.18	15.88	13.67
12	α-Ocimene	C_10_H_16_	7.62	0.00	1.29	4.78	0.00	2.60	2.85
13	3-Carene	C_10_H_16_	7.99	0.00	0.00	0.53	0.00	0.42	0.07
14	ç-Terpinene	C_10_H_16_	8.43	0.32	0.83	0.29	0.48	0.29	0.36
15	α-Pinene oxide	C_10_H_16_O	9.00	0.00	0.00	0.00	0.00	0.00	0.07
16	α-Terpinolene	C_10_H_16_	9.38	0.66	1.06	1.18	1.71	0.91	1.18
17	*cis*-Ocimene	C_10_H_16_	12.25	0.00	0.00	0.00	0.00	0.00	0.07
18	l-Menthone	C_10_H_18_O	12.39	1.37	0.00	0.00	0.00	0.00	0.00
19	Menthol	C_10_H_20_O	13.29	0.54	0.00	0.00	0.00	0.00	0.00
20	l-4-Terpineol	C_10_H_18_O	13.35	0.00	0.15	0.27	0.08	0.16	0.21
21	Cryptone (CAS)	C_9_H_14_O	13.84	0.00	0.00	0.09	0.00	0.00	0.00
22	α-Terpineol	C_10_H_18_O	14.03	1.37	1.87	2.10	0.96	2.24	2.34
23	Bornyl acetate	C_12_H_20_O_2_	17.55	0.52	0.34	0.85	0.48	0.70	0.49
24	l-Menthyl acetate	C_12_H2_2_O_2_	17.80	0.27	0.00	0.00	0.00	0.00	0.00
25	α-terpinyle acetate	C_12_H_20_O_2_	20.24	77.72	71.90	63.85	62.79	65.09	65.69
26	Isobornyl propionate	C_13_H_22_O_2_	21.36	0.09	0.00	0.00	0.08	0.00	0.00
27	Geranyl acetate	C_12_H_20_O_2_	21.66	0.00	0.38	1.06	0.35	0.48	0.58
28	α-elemene	C_15_H_24_	21.77	0.10	0.00	0.00	0.00	0.00	0.00
29	Caryophyllene	C_15_H_24_	22.96	0.21	0.00	0.00	0.00	0.00	0.00
30	Germacrene-D	C_15_H_24_	25.51	0.00	0.10	0.00	0.09	0.14	0.09
31	α-Selinene	C_15_H_24_	25.87	0.09	0.07	0.00	0.00	0.00	0.00
32	Gynoval	C_15_H_26_O_2_	26.59	0.27	0.21	0.00	0.26	0.36	0.00
33	α-Eudesmol (CAS)	C1_5_H_26_O	32.45	0.00	0.00	0.00	0.00	0.11	0.00
34	Globulol	C_15_H_26_O	32.80	0.00	0.00	0.00	0.00	0.14	0.00
35	Butylidene phthalide	C_12_H_12_O_2_	33.56	0.00	0.12	0.00	0.08	0.00	0.08
36	Ligustilide	C_12_H_14_O_2_	35.45	0.53	2.03	1.61	1.79	1.35	2.13
37	Leinoleic acid	C_18_H_32_O_2_	48.62	0.00	0.00	0.43	0.00	0.00	0.00
Total identified compounds				100.00	100.00	100.00	99.91	99.99	100.00
Total hydrocarbon compounds				17.32	23.00	29.74	33.04	29.36	28.41
Total Oxygenated compounds				82.68	77.00	70.26	66.87	70.63	71.59

1st cut and 2nd cut means the first and second cutts, respectively.

### 3.9 Simple correlation coefficients

Table 19, Table 20 shows the simple correlation coefficients among various studied characters of lovage in this study as affected with plant spacing and seeds soaking combination treatments in the first and second seasons. The symbol (*) refers to significant correlation, (^**^) means high significant correlation while (^***^) means high high significant correlation. (–) if it was observed it means negative correlation and if it is not found means positive correlation. The correlation relationship between plant parameters are in harmony with Hendawy et al. (2015) on *Mintha piperita* var. citrata and Hendawy et al. (2017) *Rosmarinus officinalis*, respectively as well as Atteya et al. (2020) on *Anthriscus cerefolium*.

**Table 19:** Simple correlation coefficients among various studied characters of lovage in this study as affected with plant spacing and melatonin concentration combination treatments in the first season.

	*1*	*2*	*3*	*4*	*5*	*6*	*7*	*8*	*9*	*10*	*11*	*12*	*13*	*14*	*15*
1	1.000														
2	0.329	1.000													
3	0.634^***^	0.387*	1.000												
4	0.280	0.979^***^	0.324	1.000											
5	0.505^**^	0.580^***^	0.843^***^	0.612^***^	1.000										
6	0.230	0.980^***^	0.354*	0.953^***^	0.542^***^	1.000									
7	−0.479^**^	0.205	−0.249	0.170	−0.180	0.308	1.000								
8	0.728^***^	0.394*	0.604^***^	0.385*	0.573^***^	0.290	−0.758^***^	1.000							
9	0.320	0.036	0.280	−0.037	0.091	0.024	0.378	−0.190	1.000						
10	0.629^***^	0.384*	0.506^**^	0.355*	0.447^**^	0.288	−0.623^***^	0.899^***^	−0.179	1.000					
11	0.024	−0.823^***^	0.124	−0.801^***^	−0.091	−0.848^***^	−0.509^**^	0.013	−0.013	−0.099	1.000				
12	−0.047	−0.829^***^	0.099	−0.818^***^	−0.133	−0.844^***^	−0.480^**^	−0.027	−0.079	−0.094	0.968^***^	1.000			
13	0.329	−0.534^***^	0.419*	−0.527^***^	0.205	−0.580^***^	−0.664^***^	0.369*	−0.068	0.250	0.867^***^	0.846^***^	1.000		
14	0.423*	0.878^***^	0.606^***^	0.898^***^	0.823^***^	0.846^***^	−0.092	0.576^***^	−0.095	0.462^**^	−0.500^**^	−0.517^**^	−0.136	1.000	
15	0.348*	0.078	0.638^***^	0.054	0.539^***^	0.119	−0.348*	0.423*	0.073	0.262	0.273	0.235	0.504^**^	0.379*	1.000

1. Plant height 2. Herb fresh weight per plant 3. Herb fresh weight  per hectare 4. Herb dry weight per plant 5. Herb dry weight per hectare 6. Roots fresh weight per plant 7. Roots fresh weight per hectare 8. Roots dry weight per plant 9. Roots dry weight per hectare 10. Chlorophyll content 11. Total phenolic 12. Antioxidant content 13. Essential oil percentage 14. Essential oil content per plant 15. Essential oil content per hectare *. Significant ^**^. High significant. **^-^**: Negative relationship.

**Table 20:** Simple correlation coefficients among various studied characters of lovage in this study as affected with plant spacing and seeds soaking combination treatments in the second season.

	1	2	3	4	5	6	7	8	9	10	11	12	13	14	15
1	1.000														
2	0.225	1.000													
3	0.487^**^	0.412*	1.000												
4	0.249	0.576^***^	0.399*	1.000											
5	0.544^***^	0.394*	0.933^***^	0.403*	1.000										
6	0.136	0.573^***^	0.302	0.982^***^	0.272	1.000									
7	−0.662^***^	0.105	−0.147	0.207	−0.265	0.335*	1.000								
8	0.774^***^	0.250	0.363*	0.428^**^	0.461^**^	0.320	−0.717^***^	1.000							
9	0.027	−0.032	0.053	−0.001	−0.087	0.036	0.451^**^	−0.265	1.000						
10	0.448^**^	0.218	0.316	0.343*	0.488^**^	0.238	−0.395*	0.693^***^	−0.084	1.000					
11	0.138	−0.357*	0.102	− 797^***^	0.129	−0.872^***^	−0.561^***^	−0.047	−0.186	−0.022	1.000				
12	0.164	−0.394*	0.132	−0.787^***^	0.161	−0.866^***^	−0.560^***^	−0.037	−0.171	−0.005	0.978^***^	1.000			
13	0.557^***^	−0.160	0.450^**^	−0.419*	0.457^**^	−0.536^***^	−0.725^***^	0.379*	−0.068	0.290	0.816^***^	0.831^***^	1.000		
14	0.513^**^	0.589^***^	0.710^***^	0.875^***^	0.725^***^	0.790^***^	−0.132	0.615^***^	−0.082	0.489^**^	−0.425^**^	−0.406*	0.045	1.000	
15	0.649^***^	0.143	0.805^***^	−0.014	0.847^***^	−0.159	−0.574^***^	0.488^**^	−0.048	0.463^**^	0.553^***^	0.583^***^	0.856^***^	0.450^**^	1.000

1. Plant height 2. Herb fresh weight per plant 3. Herb fresh weight per hectare 4. Herb dry weight per plant 5. Herb dry weight per hectare 6. Roots fresh weight per plant 7. Roots fresh weight per hectare 8. Roots dry weight per plant 9. Roots dry weight per hectare 10. Chlorophyll content 11. Total phenolic 12. Antioxidant content 13. Essential oil percentage 14. Essential oil content per plant 15. Essential oil content per hectare *. Significant ^**^. High significant. **^-^**: Negative relationship.

### 3.10 Antimicrobial activity evaluation:

Lovage volatile oil (*L. officinale*) exhibits high antibacterial and antifungal properties in the range of concentrations 75–100 µg mL^−1^ of oil concentration (Table 21).

**Table 21:** The antimicrobial activity (MBC, MFC) * of the oil extracted from the lovage plants.

Examined Bacteria and Fungi	Oil concentration µg mL^−1^				
	0	25	50	75	100
*Ralstonia solanacearum*					
(4.8 × 10^8^ CFU mL^−1^)	–	–	–	+	+
*Pectobacterium Carotovorum subsp atrosepticum*					
(4.8 × 10^8^ CFU mL^−1^)	–	–	–	+	+
*Pectobacterium Carotovorum subsp Carotovorum*					
(4.8 × 10^8^ CFU mL^−1^)	–	–	–	+	+
*Fusarium roseum* (3.0 × 10^7^ CFU mL^−1^)	–	–	–	+	+

*MBC - minimal bactericidal concentration; MFC - minimal fungicidal concentration.

## 4 Discussion

Due to the shortage of information about the response of lovage plants to agriculture practices in general. and as a now import plant under Egyptian environmental conditions this work was done to obtain the optimum combination treatment of soaking seeds in melatonin before planting and plant density for sowing lovage seeds. From the above mentioned results of combination treatments, the treatment of 15 cm between plants in row plus 75 µM melatonin gave the maximum yield of roots fresh weight, total phenolic and antioxidant content and essential oil percentage in both seasons. The treatment of 30 cm between plants in row plus soaking in 75 µM melatonin solution recorded the maximum plant height, yield of herb fresh and dry weight per ha^−1^, yield of roots dry weight of the first season, essential oil content per plant and yield per hectare in both season except plant height in the second cut of the second season. The treatment of 30 cm between plants in row plus soaking seeds in 100 µM melatonin had the maximum plant height and chlorophyll content in the second cut of the second season. The treatment of 45 cm between plants in row plus using 75 µM melatonin solution recorded the maximum herb fresh and dry weight per plant and roots fresh and dry weight per plant, chlorophyll content in both seasons except the chlorophyll content in the second cut of the second season.

Plant density is one of the important management factors that influences the yield of grown plants (Ren et al., 2017, Liu et al., 2019, Cong et al., 2019, Tian et al., 2020). The plant density influences plant growth, radiation interception, water consumption, disease resistance, weed competition and finally, crop yield (López-Bellido et al., 2005). With decreasing the plant densities the competition on natural resources such as competition for light aboveground and competition on more than 20 nutrient elements and water of irrigation belowground will be also decreased so the health and productivity of individual plants will become more better (Casper and Jackson, 1997, Tian et al., 2020). Therefore, in this study the planting space of 45 cm between plants in the same row gave the maximum herb and roots fresh and dry weight per plant and chlorophyll content, While recorded the minimum essential oil percentage, phenolic and antioxidant content compared with the other two less planting space. Moreover, with decreasing the planting space between cultivated plants the photosynthetic capacity was increased by increasing the interception of available solar radiation (Andrade et al., 2002(, exploit all field area resulting in maximizing herb and roots yield per hectare due to increasing the number of harvested plants despite of decreasing the weight of every plant alone and the secondary metabolites such as essential oil percentage, phenolic content and antioxidant content reached its maximum level with the less planting space (15 cm). From the other hand, the most suitable planting space differs from plant to the other plant and differs from parameter to the other one. Therefore, despite recording the maximum yield per hectare of fresh and dry weight of roots with the planting space of 15 cm between plants in the same row, the tallest plants, the maximum yield of herb fresh and dry weight per hectare as well as essential oil content per plant and yield per hectare of lovage plants were found with planting space of 30 cm between plants in the same row. Moreover, due to the fact of decreasing the available water of irrigation for every plant with increasing the number of plants in the same area so the degree of water deficit increase and reach the maximum level with the most narrow space. Polyphenol synthesis and accumulation in plants are generally stimulated in response to the resistance of plants to environmental stresses and particularly in water deficit stress (Nacif de Abreu and Mazzafera, 2005, Giorgi et al., 2009). These results could be explained by the fact that water deficit may be associated with an increase in secondary metabolites such as essential oil percentage, phenolic and antioxidant content through the reallocation of the assimilated carbon as plant growth is progressively reduced. In this condition melatonin is an efficient growth regulator in plants (Li et al., 2018, Arnao and Hernández-Ruiz, 2014). Soaking seeds in melatonin promotes seed emergence. As it increases the chance of survival by enhancing starch metabolism and energy supplies in response to emergence process (Zhang et al., 2015, Liu et al., 2015). After emergence, these applications of melatonin have an important role in increasing photosynthetic rates by improving plant antioxidant defense drought (Li et al., 2016a, Li et al., 2016b). In addition, the idea of soaking seeds before sowing is aimed at shortening the lag phase in emergence and to enhance seedling establishment, thereby minimizing the risk in the early vegetative growth (Sabongari and Aliero, 2004) and finally enhance growth parameter. Each crop cultivar requires a critical soaking condition for good emergence (Harris et al., 2000). So in this work, despite soaking lovage seeds in 100 µM melatonin solution gave the minimum number of days to emergence, the soaking in 75 µM melatonin before planting them accelerated emergence and also had the maximum emergence percentage compared with the other treatments including the control treatment (without soaking). These good conditions of emergence had influenced role on lovage plants growth so, using 75 µM melatonin solution for soaking seeds of lovage before planting gave the maximum mean values of all studied parameters of herb, roots and chemical constituents in this study in both cuts of both seasons, respectively compared with other treatments except for plant height and chlorophyll content of the second cut in the second season as the maximum values of it were found with 100 µM melatonin solution across all studied planting spaces. Moreover, the improving that was found in this study as a result of soaked seeds compared with unsoaked seeds is in harmony with Arshad Ullah et al. (2002) who reported that pre-sowing treatments of raya seed encourage growth of crop, reduced days to emergence, increased numbers of branches plant^−1^ and plant yield. Mozumder and Hossain (2013) found that days to first emergence decreased with increasing soaking period of *Eryngium foetidum* L. seeds. Sabongari and Aliero (2004) observed that soaking the seeds of *Lycopersicum esculentum* Mill in water before sowing them in the field improved emergence and growth compared with control seeds. Therefore, under normal condition (wide planting spacing) and water-stress conditions (narrow planting spacing) soaking seeds in melatonin solution was able to improve emergence and growth as well as all studied parameters and chemical composition. It was in harmony with all of Jiahao et al., 2019, Zhang et al., 2014, Li et al., 2016a, Li et al., 2016b, Chen et al., 2020 who found that exogenous melatonin application improved emergence and growth of seeds and plants under normal and abiotic stresses condition.

The major components of essential oil of lovage herb in the first and second cuts were β-Terpinyl acetate and β-Phellandrene as agree with Samiee et al., 2006, Mohamadi et al., 2016. It can be mentioned that the antimicrobial properties of the lovage essential oil are due to the high content of β-phellandrene, α-terpinyl acetate and Ligustilide. The mentioned compounds exhibit pronounced antimicrobial properties through mechanisms that include breaking of the cell wall and cytoplasmic membrane, reduction of the cytoplasm around the nucleus, disturbance of the lipid fraction of the plasma membrane resulting in the alteration of its permeability and the leakage of the intracellular content (Li et al., 2014, Trombetta et al., 2005).

## 5 Conclusion

From this work, it appeared that the growth of the lovage plant significantly affected by different treatments in this study. Moreover, measurements comprising of herb fresh and dry weight as well as essential oil yield per hectare showed that the combination treatment of 30 cm between putting plants in row plus soaking seeds in 75 µM melatonin solution was able to achieve the maximum values of these parameters. While the combination treatment of 15 cm between plants in row plus seeds soaking in 75 µM melatonin solution is recommended for getting the maximum yield of roots fresh and dry weight per hectare and maximum polyphenols, antioxidant content per herb in both cuts of both studied season. The maximum relative pronounce of α-terpinyle acetate were obtained with the combination treatment of 15 cm planting space and 0 µM melatonin. While the maximum relative pronounce of β-Phellandrene was recorded with the combination treatment of 30 cm plant spacing and seeds soaking solution of 75 µM melatonin

Funding

This research received no external funding.

**Institutional Review Board Statement:** Not applicable.

**Informed Consent Statement:** Not applicable.

**Data Availability Statement:** The data presented in this study are available within the article.

## Declaration of Competing Interest

The authors declare that they have no known competing financial interests or personal relationships that could have appeared to influence the work reported in this paper.
